# A Case of Drug Reaction with Eosinophilia and Systemic Symptoms

**DOI:** 10.1155/2012/705190

**Published:** 2012-08-27

**Authors:** Sally Kellett, Charles Cock

**Affiliations:** Division of Medicine, Repatriation General Hospital, Daws Road, Daw Park, SA 5041, Australia

## Abstract

Drug reaction with eosinophilia and systemic symptoms (DRESS) is characterized by fever, skin rash, hematological abnormalities, and systemic involvement such as hepatitis. DRESS usually presents 2–6 weeks after drug initiation. DRESS should be suspected on clinical grounds in the setting of the introduction of new drug therapy and is most commonly described after the introduction of aromatic anticonvulsants, allopurinol, or antiretroviral therapies. We describe here a case of DRESS due to phenytoin exposure with complete resolution on drug discontinuation. Our patient developed DRESS with a skin rash, lymphadenopathy, and markedly abnormal liver enzymes, 4 weeks after drug initiation following drainage of a brain abscess. He was initially diagnosed as having a recurrence of the abscess or sepsis of another origin. It is important to recognise the possibility of DRESS in this setting, as a good outcome depends on the immediate withdrawal of the offending drug. A mortality rate of up to 10% has been described in unrecognised cases.

## 1. Introduction

Several cutaneous reactions to medication with variable onset and manifestations occur after drug exposure [1]. These reactions are collectively known as SCAR: severe cutaneous adverse reactions to drugs. Drug reaction with eosinophilia and systemic symptoms (DRESS) is a potentially fatal drug reaction characterized by fever, a skin rash, hematological abnormalities including eosinophilia or abnormal lymphocytes and systemic involvement including hepatitis, interstitial nephritis or pneumonitis [2]. DRESS can mimic severe sepsis, autoimmune, tick borne, or viral diseases and should be considered in the differential diagnoses in these settings, particularly after the recent introduction of new drug therapies. The onset is typically 2–6 weeks (average 4 weeks) after first exposure [2]. DRESS is a syndromic manifestation of a type IV delayed hypersensitivity reaction due to reactive metabolites from drugs [3]. We now recognise several drugs beyond anticonvulsants as causative agents, and thus anticonvulsant hypersensitivity syndrome [4] should not be used to describe this syndrome, even when it occurs as a consequence of this group of drugs [2]. The RegiSCAR criteria (nonvalidated) [5] (Table 1) can be used to determine the likelihood of actual occurrence cases of DRESS. The Naranjo scale can also be used to assess the probability of an adverse drug reaction [6]. 

## 2. Case

A 47-year-old male patient presented to a metropolitan hospital with a new onset of fever, headache, right-sided weakness, and dysphasia. His background included a history of paranoid schizophrenia and cannabis abuse. The patient strongly denied the use of injectable drugs. His only medication was longstanding depot risperidone injections. He was a smoker with no known allergies. A computerized tomography (CT) of the brain revealed a 2 × 3 cm mass in the medial left frontal lobe consistent with the diagnosis of a brain abscess. Initial biochemistry and a complete blood count were normal, including liver enzyme tests. Successful neurosurgical drainage of the brain abscess was undertaken in theatre. Cultures taken from the abscess material grew *Streptococcus anginosus,* and the patient was commenced onto treatment with benzyl penicillin, according to the sensitivities. Phenytoin was initiated as a prophylactic anticonvulsant at an initial dose of 300 mg *nocte*., increased to 500 mg over 2 weeks. Anticonvulsant therapies are individualized within the neurosurgery department according to consultant preference, but the prescribed regime was in keeping with the regional clinical protocol for enteral phenytoin administration. Levels were measured and found to be below the normal range (20 umol/L (normal range 40–80 umol/L)). A week following surgery, the patient was referred to a rehabilitation unit. 

**Figure 1:**
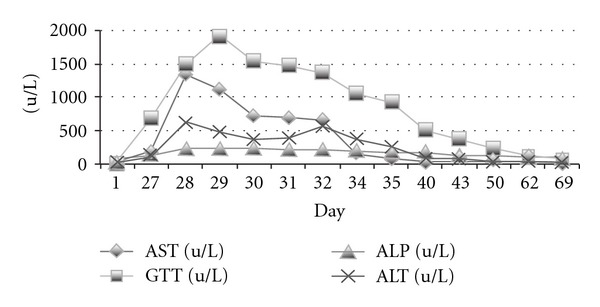
Liver enzymes show an acute rise on day 27, with gradual normalization by day 69 (42 days after the initial rise).

Three weeks after admission to the rehabilitation unit (day 27), the patient developed fever (38.4 degrees Celsius). Blood cultures were undertaken, a chest X-ray (CXR) ordered, and a CT scan of his brain repeated which showed a reduced abscess cavity compared to his previous study. A day later the patient developed erythema over his upper torso, malaise and rigors. On the recommendation of the general medical unit on take in the hospital, the patient was commenced on antibiotic therapy (vancomycin), and his biochemistry repeated. The results of the biochemistry revealed deranged liver enzymes (Figure 1) and an elevated CRP (120 mg/L (normal range <8 mg/L)). An ultrasound of the liver was ordered and further blood cultures were taken. His fever persisted and the rash worsened over the following 24 hours. Additionally he was found to have developed lymphadenopathy. The decision was made to transfer the patient to the acute general medical unit within the hospital. After discussion with the on call medical registrar, phenytoin therapy was discontinued. He was also treated with an antihistamine. The rash and temperature persisted, but all cultures remained negative and CXR clear. The liver ultrasound was essentially unremarkable. Additional autoimmune serology was negative. Serology for hepatitis A, B, and C as well as CMV, EBV, and human herpes viruses were also ordered at this point and were negative. The eosinophil count was 0.5 × 10^9^/L (N 0.02–0.05 × 10^9^/L). The patient had rhabdomyolysis with a total CK level of 3485 u/L (N < 250 u/L) and atypical lymphocytes on blood film. The benzyl penicillin was discontinued, after which the patient rapidly improved with complete resolution of fever within 24 hours and clearing of his rash. Liver enzymes remained elevated, but decreased and eventually returned to complete normality 42 days after becoming elevated (Figure 1). 

## 3. Discussion

The patient is presented as a case of DRESS. He expressed typical features of DRESS with skin rash, lymphadenopathy, raised liver enzymes, and atypical lymphocytes, three and a half weeks after commencement onto phenytoin, which is the suspected culprit drug. He did not develop overt hypereosinophilia, which is defined in DRESS as being above 0.7 × 10^9^/L [4], although his eosinophil count was raised above the normal value. He had additional evidence of rhabdomyolysis, which is described rarely in DRESS [7], but is not part of the diagnostic criteria. His RegiSCAR score was determined as 6 (see Table 1). The Naranjo score was 4 (see Table 2). His background history of a combination psychotropic and anticonvulsant drug use may serve as a predisposing factor through cytochrome P450 enzyme inhibition [7], allowing the accumulation of antigenic metabolites of a second drug metabolized via the same cytochrome P450 subenzymes. Drug therapy was discontinued rapidly in our patient following recognition of DRESS, with resolution of his symptom complex. His liver enzymes took an additional 42 days to normalize, however (Figure 1). 

Anticonvulsant therapies, particularly those with an aromatic ring structure such as carbamazepine, phenytoin, and phenobarbital have been recognised as precipitants for DRESS syndrome, in what used to be termed anticonvulsant hypersensitivity syndrome [4]. This term has been superseded in the literature by the use of the term DRESS [2, 5]. A multitude of other drugs have since been associated with this syndrome among which the most frequent are allopurinol, lamotrigine, nevirapine, sulfasalazine, and sulfonamide antibiotics [2]. The alternative term drug-induced hypersensitivity syndrome (DIHS) was coined to describe DRESS in association with human herpes virus type 6 reactivation [8, 9]. DRESS is rare, occurring in 1 : 1000 to 1 : 10000 patients prescribed anticonvulsant therapy [10] but if unrecognized, has a high mortality [2]. Death occurs in 5–10% [2]. Recognition could be delayed due to the delayed onset of the symptoms, which occur on average 4 weeks after drug initiation [2]. For this reason, primary care providers, including general practitioners, should be especially aware of the possibility of DRESS 3–6 weeks after drug initiation.

The proposed pathogenesis of DRESS relates to an abnormal immune response in a genetically susceptible individual [2, 3]. This is thought to be induced by the formation of reactive metabolites [3] and in some cases re-activation of herpes viruses 6, 7 and Epstein-Barr virus [2, 8]. In the case of aromatic anticonvulsants, toxic metabolites in the form of arene oxides are produced, which lead to a type IV hypersensitivity reaction [3]. Some patients appear to have a lack of the enzyme epoxide hydrolase, involved in the breakdown of the toxic metabolites [3]. Due to the likelihood of genetic predisposition, first degree relatives should avoid this class of drugs [2].

The disease occurs along a spectrum of severity, which depends to some degree on early recognition and discontinuation of the offending drug. A skin rash, usually an erythematous, maculopapular rash occurred in 99% of cases of DRESS described in the literature [2]. At the severe end of the spectrum, organ involvement, including liver involvement, hepatitis, is common and occurs in up to 90% of cases described in the literature [2]. Renal (9%) or pulmonary involvement (5%) is far less commonly described [2]. Organ involvement can be asymptomatic. Hematological abnormalities including hypereosinophilia >0.7 × 10^9^/L (80% of cases) and atypical lymphocytes (35% of cases) also occur commonly [2]. Lymphocytoses is another common clinical finding [2].

The major differential diagnoses include septicaemia, autoimmune diseases including vasculitides and adult Still's disease, tick borne diseases, and viral disease, including viral hepatitis and virus hemorrhagic fevers (in the appropriate context). These conditions should be excluded in all cases of suspected DRESS, either through relevant history or serology. Demonstration of herpes viruses, particularly herpes virus type 6 (HHV-6 infection), is suggestive of the diagnoses and may be a cofactor in the pathogenesis of DRESS [8]. An in vitro interferon gamma release test, where serum is exposed to different drugs taken by the patient with a subsequent increase in IFN-gamma demonstrated in the offending drug, can also be performed in cases where there is a clinical necessity to continue drug therapy. This is not yet readily available, but has been described as a useful adjunct in DRESS [11]. There is a high prevalence of cross reactivity to other aromatic anticonvulsants complicating treatment of epileptic disorders in patients with DRESS [12].

Treatment is withdrawal of the offending drug or drugs. Corticosteroids and intravenous immunoglobulins have been given in individual cases [2], but no trials of these therapies exist. The mean time for recovery is 6.4 to 9.4 weeks. Deaths described in the literature were associated with an older age and liver enzyme abnormalities [2].

Our patient fit the criteria for DRESS and showed improvement soon after discontinuation of his phenytoin. He was discharged home from the acute medical ward within a week, following drug withdrawal without the need for further rehabiltative treatment. Levetiracetam was initiated in order to replace the phenytoin. He remained on ceftriaxone at the recommendation of the infectious diseases unit and was followed up in the outpatient department with no recurrence of fever or other symptoms. The patient remained well a year following the above hospitalisation with complete normalisation of inflammatory markers and liver enzymes. 

## Figures and Tables

**Table 1:** RegiSCAR criteria for the likelihood of DRESS syndrome.

	No	Yes	Unknown
(1) Fever > 38.5^°^C	−1	0	−1
(2) Enlarged lymph nodes (>2 sites, >1 cm)	0	1	0
(3) Atypical lymphocytes	0	1	0
(4) Eosinophilia	0		0
0.7–1.49 × 10^9^/L or 10–19.9%		1	
>1.5 × 10^9^/L or ≥20%		2	
(5) Skin rash	0		0
Extent > 50%	0	1	0
At least 2 of: edema, infiltration, purpura, scaling	−1	1	0
Biopsy suggestive of DRESS	−1	0	0
(6) Internal organ involvement	0		0
One		1	
2 or more		2	
(7) Resolution in >15 days	−1	0	−1
(8) At least 3 investigations negative for alternative cause	0	1	0

Final score: <2 No case; 2-3 possible case, 4-5 probable case; >5 definite case.

**Table 2:** Naranjo score for the likelihood of an adverse drug reaction.

	Yes	No	Unknown
(1) Previous conclusive reports on this reaction	1		
(2) Did the adverse event appear after suspected drug given?	2		
(3) Did the adverse reaction improve when the drug was discontinued or a specific antagonist given?	1		
(4) Did the adverse reaction appear when the drug was readministered?			0
(5) Are there alternative causes that could have caused the reaction?	−1		
(6) Did the reaction reappear when a placebo was given?			0
(7) Was the drug detected in any body fluid in toxic concentrations?		0	
(8) Was the reaction more severe when the dose was increased or less severe when decreased?		0	
(9) Did the patient have a similar reaction to the same or similar drugs in any previous exposure?		0	
(10) Was the adverse event confirmed by any objective evidence?	1		

Final score: >9 Definite ADR; 5–8 probable ADR; 1–4 possible ADR; <0 doubtful ADR.

## References

[1] Roujeau JC, Allanore L, Liss Y, Mockenhaupt M (2009). Severe cutaneous adverse reactions to drugs (SCAR): definitions, diagnostic criteria, genetic predisposition. *Dermatologica Sinica*.

[2] Cacoub P, Musette P, Descamps V (2011). The DRESS syndrome: a literature review. *American Journal of Medicine*.

[3] Shear NH, Spielberg SP (1988). Anticonvulsant hypersensitivity syndrome. In vitro assessment of risk. *The Journal of Clinical Investigation*.

[4] Vittorio CC, Muglia JJ (1995). Anticonvulsant hypersensitivity syndrome. *Archives of Internal Medicine*.

[5] Kardaun SH, Sidoroff A, Valeyrie-Allanore L (2007). Variability in the clinical pattern of cutaneous side-effects of drugs with systemic symptoms: does a DRESS syndrome really exist?. *British Journal of Dermatology*.

[6] Naranjo CA, Busto U, Sellers EM (1981). A method for estimating the probability of adverse drug reactions. *Clinical Pharmacology and Therapeutics*.

[7] Mahapatra S, Belgrad JL, Adeoye MA (2011). Psychotropic drug-related eosinophilia with systemic symptoms after acute caffeine ingestion. *Pediatrics*.

[8] Suzuki Y, Inagi R, Aono T, Yamanishi K, Shiohara T (1998). Human herpesvirus 6 infection as a risk factor for the development of severe drug-induced hypersensitivity syndrome. *Archives of Dermatology*.

[9] Kano Y, Shiohara T (2009). The variable clinical picture of drug-induced hypersensitivity syndrome/drug rash with eosinophilia and systemic symptoms in relation to the eliciting drug. *Immunology and Allergy Clinics of North America*.

[10] Fiszenson-Albala F, Auzerie V, Make E (2003). A 6-month prospective survey of cutaneous drug reactions in a hospital setting. *British Journal of Dermatology*.

[11] Ben-Ari K, Goldberg I, Shirazi I (2008). An unusual case of DRESS syndrome. *Journal of Dermatological Case Reports*.

[12] Mansur AT, Pekcan YS, Göktay F (2008). Anticonvulsant hypersensitivity syndrome: clinical and laboratory features. *International Journal of Dermatology*.

